# Long Noncoding RNA GAS5 in Breast Cancer: Epigenetic Mechanisms and Biological Functions

**DOI:** 10.3390/ijms22136810

**Published:** 2021-06-24

**Authors:** Elena A. Filippova, Marina V. Fridman, Alexey M. Burdennyy, Vitaly I. Loginov, Irina V. Pronina, Svetlana S. Lukina, Alexey A. Dmitriev, Eleonora A. Braga

**Affiliations:** 1 Institute of General Pathology and Pathophysiology, 125315 Moscow, Russia; p.lenyxa@yandex.ru (E.A.F.); burdennyy@gmail.com (A.M.B.); loginov7w@gmail.com (V.I.L.); zolly_sten@mail.ru (I.V.P.); sveta_sergeevna349@mail.ru (S.S.L.); 2 Vavilov Institute of General Genetics, Russian Academy of Sciences, 119991 Moscow, Russia; marina-free@mail.ru; 3 Engelhardt Institute of Molecular Biology, Russian Academy of Sciences, 119991 Moscow, Russia; alex_245@mail.ru

**Keywords:** lncRNA, GAS5, breast cancer, apoptosis, metastasis, epigenetic mechanisms, ceRNA, methylation, sensitivity to chemotherapy

## Abstract

Long noncoding RNAs (lncRNAs) have been identified as contributors to the development and progression of cancer through various functions and mechanisms. LncRNA GAS5 is downregulated in multiple cancers and acts as a tumor suppressor in breast cancer. GAS5 interacts with various proteins (e.g., E2F1, EZH2, and YAP), DNA (e.g., the insulin receptor promoter), and various microRNAs (miRNAs). In breast cancer, GAS5 binds with miR-21, miR-222, miR-221-3p, miR-196a-5p, and miR-378a-5p that indicates the presence of several elements for miRNA binding (MREs) in GAS5. Mediated by the listed miRNAs, GAS5 is involved in the upregulation of a number of mRNAs of suppressor proteins such as PTEN, PDCD4, DKK2, FOXO1, and SUFU. Furthermore, the aberrant promoter methylation is involved in the regulation of *GAS5* gene expression in triple-negative breast cancer and some other carcinomas. GAS5 can stimulate apoptosis in breast cancer via diverse pathways, including cell death receptors and mitochondrial signaling pathways. GAS5 is also a key player in the regulation of some crucial signal pathways in breast cancer, such as PI3K/AKT/mTOR, Wnt/β-catenin, and NF-κB signaling. Through epigenetic and other mechanisms, GAS5 can increase sensitivity to multiple drugs and improve prognosis. GAS5 is thus a promising target in the treatment of breast cancer patients.

## 1. Introduction

The recent steady increase in the rate of morbidity due to various cancers has heightened the urgency to determine mechanisms of carcinogenesis [1]. Malignant transformation involves molecular, genetic and epigenetic factors [2]. The early detection of cancer, the search for potential therapeutic targets, and the identification of factors that can increase sensitivity to chemotherapy are necessary for successful treatment and are paramount issues in cancer research [2]. One crucial research goal is the identification of new diagnostic and prognostic biomarkers that could become the basis for novel and effective treatment strategies.

Advances in high-throughput sequencing methodologies have enabled the identification of biomolecules termed noncoding RNAs (ncRNAs). Several types of ncRNAs include long ncRNAs (lncRNAs). LncRNAs are a group of RNA molecules that exceed 200 nucleotides in length. Along with regulatory microRNAs (miRNAs), lncRNAs are involved in many biological processes and are crucial in regulating the expression of key genes [3]. Once thought to have no function, lncRNAs are now recognized as the most important regulators of the progression and metastasis of various cancers.

Research has also indicated that lncRNAs can serve as diagnostic and/or prognostic biomarkers for different types of cancer [4,5]. Many lncRNA genes are oncogenes whose expression levels are increased in tumors compared to matched non-cancerous tissues from the same patient [2]. In contrast, another group of genes that encode suppressor lncRNAs are downregulated in tumors and suppress tumor growth and progression [2].

In most cancers, the growth arrest-specific 5 (GAS5) lncRNA can suppress the expression of many oncogenes, including a number of miRNAs and lncRNAs, as well as activate other tumor suppressors (see reviews in [6,7,8,9,10,11,12,13]). GAS5 expression is reduced in diverse cancers, including gastric cancer, melanoma, esophageal squamous cell carcinoma, non-small cell lung cancer, ovarian cancer, cervical cancer, gliomas, osteosarcoma, pancreatic cancer, bladder cancer, renal cell carcinoma, papillary thyroid carcinoma, neuroblastoma, endometrial cancer, and hepatocellular carcinoma [13], with only some types of cancer exhibiting inconsistent results [9]. The decreased expression of lncRNA GAS5 has been correlated with the clinical characteristics of the tumor, such as metastasis to the lymph nodes, tumor recurrence, decreased survival rates, and resistance to chemotherapy [9,10,11,12,13,14]. The experimental increase in GAS5 expression stimulates apoptosis and inhibits proliferation, invasion, and epithelial-mesenchymal transition [7,15,16,17]. GAS5 is contained in the exosomes produced by healthy tissues and its content decreases in lung cancer, providing the basis for a non-invasive diagnosis [18,19]. With its generally oncosuppressive actions, the possible mechanisms of GAS5 that influence oncogenesis and other cellular processes are complex and diverse. Moreover, the conditions in humans may differ from those in models, primarily mice models [12].

## 2. Biogenesis, Biological Processes, and Molecular Mechanisms of lncRNA GAS5 in Cells and Tumors

The *GAS5* gene was originally isolated from mouse genomic DNA. The designation of the gene reflected its presence in the cytoplasm of growth-arrested cells [7,20]. *GAS5* belongs to the 5′-terminal oligopyrimidine (5′-TOP) gene family, which also includes genes that encode all ribosomal proteins, the elongation factors of protein synthesis, and many other genes not associated with ensuring the functional activity of ribosomes [21]. This locus is located on chromosome 1q25.1 and consists of 12 exons with a short open reading frame (ORF). Some portions of introns with highly conserved regions are loci of small nucleolar RNAs (snoRNAs) [7,22]. LncRNA GAS5 has alternative transcriptional start sites and several splice forms [12]. This factor may explain, in particular, the unusual activities of lncRNA GAS5 (e.g., anti-apoptotic action) [23]. The gene has a promoter CpG island, in which the level of methylation determines its activity in cancer and non-cancer tissues [24,25].

**Figure 1:**
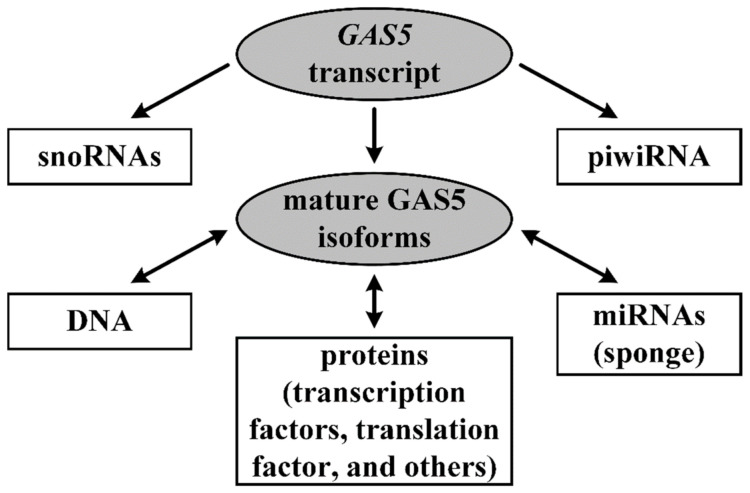
Functions of the *GAS5* gene in a cell. Straight arrows—biogenesis; double-headed arrows—interactions.

The remainder of this subsection provides a brief overview of the main aspects of *GAS5* cell functions and their relationship with certain structural elements of the gene and its lncRNA (Figure 1).

### 2.1. Protein Synthesis

Protein synthesis from ORFs in the mouse *GAS5* gene has not been observed. Furthermore, the short ORFs of mouse and human *GAS5* genes are defective in the sense that they contain a stop codon located at a significant distance from the end of the lncRNA. Nevertheless, indirect arguments indicate that, in humans, these ORFs can be translated under certain conditions [12]. The defective nature of ORFs enables regulated nonsense-mediated RNA decay, so translation can lead to the destruction of this lncRNA after the first round [26,27]. Since GAS5 belongs to the 5′-TOP family, the initiation of GAS5 translation depends on the mammalian target of rapamycin (mTOR) [28]. In this mechanism, TOR reduces the level of this lncRNA in cells [6,12]. Therefore, the use of mTOR inhibitors increases the expression of GAS5. For several cancers, the suppression of GAS5 and mTOR expression has been shown to be reciprocal [27,29]. It was suggested that these ORFs can “collect” ribosomes on themselves without protein synthesis, acting as a “sponge” for ribosomes [12].

### 2.2. SnoRNAs

SnoRNAs encoded in *GAS5* introns can also influence a number of processes, including those associated with carcinogenesis. In the case of an increase in p53 expression caused by DNA damage, snoRNAs are assumed to determine the responses to these events in colorectal cancer [30]. The overexpression of SNORD76 can inhibit the growth of glioblastoma in vitro and in vivo. A decrease in the level of SNORD76 correlates better with cancer stage, according to the World Health Organization’s classification, than a decrease in the expression of the host gene, *GAS5* [31].

### 2.3. P-Element Induced Wimpy Testis (Piwi)

SNORD75, which is encoded in one of the *GAS5* introns, is a precursor of Pi-sno-75, which binds to PIWIL1 and PIWIL4 proteins. The increased expression of Pi-sno-75 in breast cancer leads to histone modifications in the promoter of the gene of tumor necrosis factor-related apoptosis-inducing ligand (TRAIL). These modifications are dependent on PIWIL1 and PIWIL4 and cause an increase in TRAIL expression [32].

### 2.4. Interaction of lncRNA GAS5 with DNA

GAS5 binds to the promoter of the insulin receptor gene and increases the expression of the receptor [33]. Since the efficiency of this process can be affected by the protection of lncRNA from nonsense-mediated RNA decay, which occurs in the cytoplasm, GAS5 should be relatively easily transferred from the nucleus to the cytoplasm and back.

### 2.5. Interaction of lncRNA GAS5 with miRNAs

In different types of cancer, GAS5 interacts via a sponge mechanism with miRNAs, including miR-18a, 21, 23a, 106b, 135a/b, 182, 196a, 205, 221, and 222 (see the review in [14]). These interactions occur mainly with the exon regions of GAS5, including those located far from the 3′-end [12]. A significant portion of the suppressive effect of GAS5 is explained by the inhibited activity of these miRNAs. The important role of these mechanisms is emphasized by the presence of systems with positive feedback that are capable of “switching” the mode of cell functioning. For example, GAS5 and miR-21 are characterized by mutual repression in breast cancer cells [34]. GAS5 binds to miR-196a, increasing the expression of its target FOXO1 (Forkhead Box Protein O1). In turn, FOXO1 promotes the transcription of GAS5, thus forming a positive feedback loop [35]. Unfortunately, it has not yet been shown whether a similar positive feedback loop exists for breast cancer.

### 2.6. Interaction of lncRNA GAS5 with Proteins

There are several aspects of interaction of lncRNA GAS5 with proteins. The sequence near the 3′-end (exon 12) of most GAS5 isoforms operates as a so-called riborepressor. The sequence forms a highly stable stem-loop structure that can successfully compete with the glucocorticoid response element in DNA for binding to glucocorticoid receptors. Binding can occur with the androgen receptor, glucocorticoids, mineralocorticoids, and progesterone but not with α-estrogen [36]. The riborepressor affects the expression of genes both induced and suppressed by glucocorticoids. One of the genes induced by glucocorticoids is Baculoviral IAP Repeat Containing 3 (BIRC3, also termed cIAP2), a member of the IAP family of proteins that inhibit apoptosis. Prevention of this gene’s induction is partially associated with the proapoptotic effect of GAS5 [36]. GAS5 interacts with E2F1 (E2F Transcription Factor 1) and enhances its binding to the P27Kip1 promoter, which leads to cell cycle arrest in prostate cancer [37]. In epithelial ovarian cancer, GAS5 interacts with E2F4 (E2F Transcription Factor 4), recruiting E2F4 to the PARP1 promoter [38]. In gastric cancer, GAS5 binds to the YBX1 (Y-Box Transcription Factor) protein. This binding process stabilizes YBX1, resulting in more efficient activation of p21 and cell cycle arrest. GAS5 binding is significantly reduced due to the deletion of exon 12 in GAS5 [39]. GAS5 in glioma cells directly interacts with EZH2 (Enhancer of Zeste 2 Polycomb Repressive Complex 2 Subunit). This interaction results in the formation of polycomb repressive complex 2 (PRC2), decreased levels of DNA methyltransferases, methylation of the miR-424 gene promoter, and an increase in miR-424 expression [40]. GAS5 inhibits the progression of colorectal cancer through its direct interactions with the WW-domain of YAP (Yes1 Associated Transcriptional Regulator), thereby facilitating YAP translocation from the nucleus to the cytoplasm and promoting phosphorylation and ubiquitin-mediated degradation; this interaction takes place at 262–480 bp [41]. GAS5 also interacts with eIF4E (Eukaryotic Translation Initiation Factor 4E) through two RNA-binding domains of the latter. As a result, the binding of c-Myc to the polysomes is disrupted, and the translation activity decreases [42] for lymphoma cell lines. GAS5 may also interact directly with cyclooxygenase-2 (COX-2) [43] and glucose-6-phosphate dehydrogenase (G6PD) [44].

Many of the functional activities of GAS5 and its gene are carried out independently of each other and are associated with different regions of the molecule [45]. Polymorphic variant characteristic of different regions of *GAS5* can also affect different processes.

## 3. GAS5 in Epigenetic Regulation in Breast Cancer

### 3.1. CpG Methylation May Suppress GAS5 Expression

LncRNA is an epigenetic factor that can be simultaneously regulated by another epigenetic factor; for example, by changing the methylation level of the encoding gene. CpG methylation of the promoter region of the *GAS5* gene was reportedly increased in triple-negative breast cancer (TNBC) tissues and TNBC cell lines, while the expression of GAS5 was suppressed in these tumor cells [46]. The inhibitory effect of DNA methylation on the expression of the *GAS5* gene was demonstrated in other cancers, such as gastric cancer and cervical cancer [24,25]. There is no doubt that methylation of the promoter CpG island is at least partly involved in the downregulation of *GAS5* gene expression in various cancers, including breast cancer.

### 3.2. LncRNAs as Competitive Endogenous RNAs

LncRNAs are involved in a variety of processes, from the modification of histones and the effect on chromatin remodeling to the regulation of genes at the post-transcriptional level via interactions with mRNA or miRNA. The proposed and widely accepted mechanism of gene regulation is termed the competitive endogenous RNA (ceRNA) model. In this model, lncRNA competes with protein-coding mRNAs to enable miRNA binding. LncRNA can interact with the same miRNA segments involved in the binding of target mRNA. Over a decade ago, it was hypothesized that transcripts of protein-coding genes, pseudogenes, miRNAs, and lncRNAs are involved in a complex network of interactions mediated by miRNA response elements (MRE) [47]. Much less frequently, the interaction of lncRNA with mRNA is direct. For example, the direct binding of lncRNA WDR7-7 (WD Repeat Domain 77) to the mRNA of the G-protein coupled estrogen receptor 30 (GPR30) gene was previously described in cell cultures of breast cancer [48]. Moreover, due to the ceRNA mechanism, lncRNAs, mRNAs, and miRNAs form large-scale regulatory networks in cancer cells, including breast cancer [49,50]. The collective data related to lncRNAs have added new layers to these networks and deepened our understanding of the mechanisms of tumorigenesis.

### 3.3. miR-21 as a Mediator in the GAS5 Functions in the ceRNA Model

The involvement of lncRNA GAS5 in these mechanisms was first demonstrated for miR-21 in 2013 [34]. The GAS5 level in non-malignant breast MCF-10A cells was found to be higher than that in MCF-7 or MDA-MB-231 breast cancer cells, detected using qRT-PCR. A negative correlation was established between the expression levels of GAS5 and miR-21 in MCF-7 and MDA-MB-231 cells and clinical breast cancer samples. The mutual inhibitory effect of GAS5 and miR-21was revealed in experiments on the transfection of breast cancer cell lines. GAS5–siRNAs increased the miR-21 level, while the ectopic expression of GAS5 suppressed miR-21. Wild-type miR-21 was able to suppress GAS5 by over 50%. In the 3′-region of GAS5, a complementarity site with the miR-21 sequence was identified [34]. In the same study, RNA was successfully immunoprecipitated using antibodies against Argonaute RNA-induced silencing complex (RISC) catalytic component 2 (AGO2), a key component of RISC and biotin-labeled GAS5 and miR-21 probes. A pull-down experiment detected both GAS5 and miR-21 in the RISC complex, confirming their direct binding [34]. The authors also confirmed the suppressor function of lncRNA GAS5 in breast cancer.

In the same study, GAS5 sensitized tumor cells to ultraviolet (UV) irradiation and the chemotherapy drug doxorubicin. Additional data suggested that GAS5 can suppress cell invasion by regulating phosphatase and tensin homologs (PTEN) and Programmed cell death protein 4 (PDCD4) mediated by miR-21 [34]. The effect of GAS5 on cell growth was studied using 3-(4,5-dimethylthiazol-2-yl)-2,5-diphenyltetrazolium bromide and colony-formation assays after the transfection of GAS5. GAS5 enhanced UV- and doxorubicin-induced cell growth inhibition, likely by regulating apoptosis [34]. The authors also demonstrated that GAS5 significantly suppressed cell invasion in MDA-MB-231, as GAS5–small interfering RNA increased cell invasion up to 60% in MDA-MB-231 cells. This study demonstrated the participation of GAS5 in inhibiting the growth and invasion of breast cancer cells by binding oncogenic miR-21 and activating its targets, such as PTEN and PDCD4.

A 2016 study noted that GAS5 was among the top 15 downregulated lncRNAs of HER2-positive breast cancer [51]. The authors found that the lower expression level of GAS5 in breast cancer was associated with an advanced TNM stage, histological grading, metastasis, poor survival, and trastuzumab resistance. In addition, the authors showed that the inhibitory effect of GAS5 on miR-21 expression in HER2-positive breast cancer cell lines led to activation of the miR-21 target, PTEN mRNA, and establishment of the GAS5/miR-21/PTEN axis in breast cancer. Moreover, GAS5 and PTEN levels were positively correlated in breast cancer cell lines. Interestingly, the inhibition of mTOR by rapamycin reportedly increased the expression of GAS5. The activation of mTOR was accompanied by decreased expression of GAS5 and PTEN (mediated by miR-21), leading to an increase in the resistance of breast cancer patients to trastuzumab [51].

### 3.4. miR-221/222 as Mediators in GAS5 Functions in the ceRNA Model

The use of MCF-7 and tamoxifen-resistant MCF-7R cell lines established the role of lncRNA GAS5 in increasing tamoxifen sensitivity in MCF-7R cells in vitro and in vivo in nude mice [52]. This increased sensitivity was reduced through the binding of GAS5 with oncogenic miR-222, the target of which was the PTEN protein mRNA. RNA immunoprecipitation (RIP) and dual-luciferase reporter gene assays were used to demonstrate the direct binding of GAS5 with miR-222, as well as the positive correlation between the expression of PTEN and GAS5 [52]. In this way, a new axis of interactions for lncRNA GAS5 in breast cancer was revealed: GAS5/miR-222/PTEN. By suppressing miR-222, GAS5 can increase the efficiency of tamoxifen in breast cancer.

A recent study analyzed the response of breast cancer patients to treatment with adriamycin (ADR) and the genotoxic anthracycline drug epirubicin. The results revealed that GAS5 expression was considerably downregulated in non-responders compared to that in the responders [53]. Plasmid (pcDNA-GAS5)-overexpressing GAS5 increased MCF-7/ADR apoptosis and reversed breast cancer cell chemoresistance to ADR-based chemotherapy in vitro. The bioinformatics analysis revealed complementary sites between miR-221-3p and GAS5. The dual-luciferase reporter and RIP assays confirmed the direct binding between miR-221-3p and GAS5 in MCF-7/ADR breast cancer cells. The dual-luciferase reporter assay also showed direct binding between miR-221-3p and dickkopf 2 (DKK2). These findings established a new axis (GAS5/miR-221-3p/DKK2) that might increase the sensitivity of breast cancer patients to ADR treatment. Furthermore, plasmid pcDNA-GAS5 increased DKK2 expression and decreased β-catenin, c-Myc, cyclin D1, and ABCB1 expressions. Therefore, GAS5 activates the Wnt/β-catenin signaling pathway via the GAS5/miR-221-3p/DKK2 axis and may be used to enhance the sensitivity of breast cancer patients to ADR treatment [53].

### 3.5. miR-196a-5p as a Mediator in GAS5 Functions in the ceRNA Model

In clinical samples of triple-negative breast cancer (TNBC), the most aggressive type of breast cancer, the expression of GAS5 was reduced in tumors, and a further decrease in the level of this lncRNA with increasing grade was observed in tumors of patients with metastases, late-stage cancer, and poor overall survival. GAS5 levels were decreased in breast cancer cell lines—most significantly in TNBC cell lines MDA-MB-231 and MDA-MB-468 [54]. Given that GAS5 enhances apoptosis and inhibits breast cancer cell proliferation, the same study assessed the effect of GAS5 on apoptosis and cell proliferation in TNBC cell lines. The overexpression of GAS5 significantly increased the apoptosis rate of MDA-MB-231 and MDA-MB-468 cells. After the overexpression of GAS5 in MDA-MB-231 cells, qRT-PCR revealed that miR-196a-5p exhibited the most significant reduction among the five potential target miRNAs. These findings indicated the negative relationship between GAS5 and miR-196a-5p expression among TNBC patient samples. The dual-luciferase assay confirmed the direct binding of miR-196a-5p with GAS5. The collective findings indicate that GAS5 enhances apoptosis and inhibits proliferation, invasion, and metastasis, while miR-196a-5p instead stimulates TNBC progression. Thus, GAS5 can suppress TNBC progression by competitively binding miR-196a-5p. In addition, the inhibitory effect of GAS5 on miR-196a-5p affects the downstream FOXO1/PI3K/AKT signal pathway [54]. These data provide support for the GAS5/miR-196a-5p/FOXO1 axis.

### 3.6. miR-378a-5p as a Mediator in GAS5 Functions in the ceRNA Model

Other authors demonstrated the direct binding of GAS5 with miR-378a-5p using RIP and luciferase assays [55]. Flow cytometry demonstrated the involvement of miR-378a-5p in GAS5-promoted apoptosis in MDA-MB-231 and BT549 cells. The same authors used a luciferase assay to detect the direct interaction of miR-378 with its potential target, the suppressor of fused protein (SUFU), in MDA-MB-231 TNBC cells.

In this study, paclitaxel (PTX) and cisplatin (CIS), two commonly used clinical chemotherapy drugs that form complexes with GAS5, were tested on TNBC cell lines [55]. The expression of GAS5 was increased in PTX-resistant MDA-231-PTX and BT549-PTX cells, as well as in CIS-resistant MDA-231-CIS and BT549-CIS, compared to the parental cells. The expression levels of GAS5 in the tumor tissues of 156 patients treated with PTX and CIS were also determined. The 5-year survival rates of patients in the GAS5-low group were also much lower than those in the GAS5-high group. The results confirmed that lncRNA GAS5 can enhance survival and tumor sensitivity to PTX and CIS. These data agree with the influence of GAS5 on tamoxifen resistance in breast cancer [52] and ADR-based therapeutic resistance [53].

Moreover, as shown in the cited work [55], the overexpression of SUFU substantially restored the sensitivity of MDA-231-PTX and MDA-231-CIS cells to PTX and CIS, respectively, confirming that SUFU might be a target for miR-378-mediated apoptosis induced by lncRNA GAS5. These effects were also confirmed in vivo using nude mice. Thus, GAS5 induces apoptosis and enhances the sensitivity of TNBC cells to PTX and CIS, at least partially through the lncRNA GAS5/miR-378a-5p/SUFU axis.

**Figure 2:**
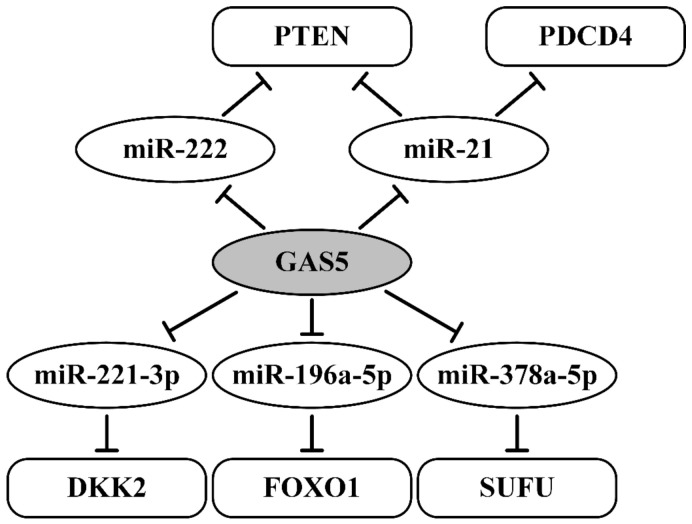
Regulating axes of lncRNA GAS5 in breast cancer. Blunt arrows indicate inhibitory effects. The following references were used: [34,51,52,53,54,55].

All of the aforementioned information supports the conclusion that GAS5 promotes apoptosis, suppresses metastasis, enhances sensitivity to a number of drugs, and increases the survival rate of breast cancer patients, including TNBC, through the participation of some mechanisms according to the ceRNA model (Figure 2). Furthermore, these data reveal several sites (i.e., MREs) that can bind various miRNAs (miR-21, miR-222, miR-221-3p, miR-196a-5p, and miR-378a-5p) in GAS5 in breast cancer. These data also indicate multiple functions of GAS5 as a ceRNA—involvement in the upregulation of a number of mRNAs of suppressor proteins, including PTEN, PDCD4, DKK2, FOXO1, and SUFU. Moreover, suppressive lncRNA GAS5 and regulating suppressive proteins play a critical role in enhancing the sensitivity of breast cancer patients to multiple drugs.

## 4. GAS5 in the Biological Processes and Pathways in Breast Cancer

### 4.1. GAS5 and Apoptosis

The very first studies on the effect of GAS5 on apoptosis in various mammalian cell lines [56] already reported that some variants of GAS5 transcripts became sensitive to various agents that can stimulate apoptosis (e.g., to chemotherapy) and that apoptosis was induced in some cell lines. The induction of apoptosis was also found to be caused by transcripts not containing an ORF [56]. The authors attributed these observations to the influence of snoRNAs encoded in the introns of *GAS5*. Subsequently, however, the main role in the induction of apoptosis was determined to be the interactions of GAS5 with various miRNAs, as well as its action as a riborepressor. Even for TNBC cells, for which therapy is the most problematic, increased GAS5 expression increased the level of apoptosis and sensitivity to chemotherapy [46].

In summary, the pathways by which GAS5 can stimulate apoptosis in breast cancer are diverse and include both external pathways—involving cell death receptors (DRs) mediated by caspase 8—and the internal mitochondrial signaling pathway (involving the release of cytochrome c and the activation of caspase 9).

#### 4.1.1. Riborepressor and cIAP2

A stem-loop sequence constituting the GAS5 hormone response element mimic was sufficient to induce apoptosis [57]. This induction of apoptosis was achieved by influencing the induction and repression of certain hormone-induced genes. As previously mentioned, the riborepressor mechanism allows GAS5 to prevent the induction of the cIAP2 gene, which suppresses apoptosis [36]. The cellular inhibitor of apoptosis protein 2 (cIAP2) binds to tumor necrosis factor associated factor 2 (TRAF2). This factor recruits the IAP2 protein to TNF receptor 1- and 2-associated complexes, which suppresses caspase-8 activation and death-receptor-mediated apoptosis [58].

cIAP2 is reportedly expressed in normal breast tissue more strongly than in cancer samples [59]. However, recent studies demonstrated a significant increase in cIAP2 expression in breast cancer, an increase in expression at later stages, and a negative correlation with survival [60]. Baculoviral IAP Repeat Containing 3 (BIRC3, also termed cIAP2) is currently believed to be involved in the mechanisms of breast cancer progression (e.g., [61]). In addition, an increase in BIRC3 expression is associated with doxorubicin resistance [62].

#### 4.1.2. TRAIL

TRAIL expression is stimulated by one of the products of the *GAS5* gene [32]. TRAIL is a member of the TNF superfamily of pro-apoptotic protein ligands (also including TNF and cluster of differentiation (CD)95L (FasL/APO-1L)). In cancer cells, including breast cancer cells, TRAIL is underexpressed, and stimulation of its expression induces apoptosis. TRAIL-induced apoptosis is triggered through the activation of death receptors, specifically DR4 and DR5. Subsequent processes include the activation of pro-caspases 8 and 10, followed by activation of caspase 3. The activation of caspase 3, in turn, leads to the activation of either the external pathway (mediated by caspase 8) or the internal pathway (release of cytochrome c and activation of caspase 9) [63].

In breast cancer, TRAIL is an important therapeutic target that enables therapeutic influence on the tumor by inducing apoptosis, as well as increasing its sensitivity to chemotherapy. Breast cancer cells with acquired resistance to endocrine therapy exhibited increased sensitivity to TRAIL and reduced the formation of cancer stem cells in TRAIL’s presence [64]. TRAIL induces apoptosis in triple-negative breast cancer cells with a mesenchymal but not an epithelial phenotype [65]. However, the effectiveness of the apoptotic action of TRAIL depends on many internal and external causes [63,66], and there is evidence that the effect of death receptors on apoptosis and cancer cell survival is ambiguous [66].

#### 4.1.3. PTEN, PDCD4, BIM, and SUFU

The interactions of GAS5 with miRNAs and the effects on the expression of their targets were largely covered in the previous section.

Increased expression of PTEN, the target of miR-21, induces apoptosis in breast cancer cells and decreases cell resistance to chemotherapy through the activation of mitochondrial-based intrinsic apoptosis pathways, which enhances the expression of caspases 3 and 9 [67]. Another miR-21 target, PDCD4, also induces apoptosis in breast cancer [68]. One of the possible mechanisms of action for this process is the binding of an internal ribosome entry site of the anti-apoptotic protein X chromosome-linked inhibitor of apoptosis (XIAP) and B-cell lymphoma-extra-large (Bcl-XL) mRNAs and inhibition of their translation [69]. XIAP binds to caspase-3, caspase-7, and caspase-9 and inhibits their activity [58]. The action of Bcl-XL is also associated with mitochondrial mechanisms of apoptosis activation, but it has both pro- and anti-apoptotic isoforms [70].

Another target of GAS5 is miR-221 [53], for which the mechanisms of suppression of apoptosis induced by various types of chemotherapy have been investigated. miR-221 can induce trastuzumab resistance by acting on its target PTEN in HER2-positive breast cancer [71]. Knockdown of miR-221 leads to cisplatin-induced apoptosis, presumably due to an increase in BIM expression, which, by binding to Bax and Bak, leads to mitochondrial dysfunction [72].

For miR-378a-5p, one more target of GAS5, an apoptotic effect on TNBC and an increase in sensitivity to paclitaxel are associated with miR-378a-5p suppression by SUFU [55]. SUFU is a negative regulator of the Hedgehog signaling pathway that regulates cell fate and survival [73].

### 4.2. GAS5 and Breast Cancer Metastasis

Many studies have addressed the effect of GAS5 in breast cancer on apoptosis and sensitivity to chemotherapy. However, few studies have examined the effect of GAS5 on the various processes associated with breast cancer metastasis. Only one study provided evidence that GAS5 knockdown induces epithelial-mesenchymal transition in breast cancer cells in vitro [74]. The weakening of the invasive properties of TNBC cells due to the suppressive effect of GAS5 on miR-196a and an increase in FOXO1 expression have also been described [54]. However, the importance of invasion for metastasis in breast cancer has been disputed (see the review in [75]). There are also some indirect arguments for the influence of GAS5 on breast cancer metastasis. Thus, PTEN, the target of a number of miRNAs regulated by GAS5, is an indicator of the invasion and metastasis of breast cancer. Loss of PTEN expression may contribute to lymphatic metastasis [76].

How significant the effect of GAS5 is on metastasis in breast cancer remains unanswered. This effect has also been observed for many other types of cancer [25,77,78,79,80,81], sometimes due to effects on the same target, miR-221 [82].

### 4.3. GAS5 and Autophagy in Breast Cancer

Autophagy (“self-eating”) describes a catabolic process. Autophagy is both a death mechanism and a cell survival mechanism. At the onset of tumor formation, autophagy limits tumor growth by preventing the accumulation of misfolded proteins, organelles, and reactive oxygen species. Stimulating autophagy may be particularly important for inducing cell death in apoptosis-resistant breast cancer cell lines. Autophagic and apoptotic cell death can regulate each other and, under certain circumstances, compensate for each other. A decrease in one of the pathways can lead to the activation of another mechanism of cell death. Conversely, the induction of autophagy may promote the survival of cancer cells by protecting breast cancer cells under stress conditions such as hunger and hypoxia, promoting metastasis, and increasing drug resistance [83]. The known and postulated mechanisms of GAS5′s influence suggest that the expression of GAS5 stimulates both apoptosis and autophagy. However, few studies have specifically addressed this point.

#### 4.3.1. ULK 1/2

In breast cancer, the expression of GAS5 correlates with the expression of unc-51-like autophagy activating kinases (ULK) 1/2 (the examined collection, unfortunately, did not contain TNBC samples). Upon in vitro upregulation, GAS5 stimulates ULK1/2, thereby enhancing autophagy and inhibiting cell proliferation. Increased sensitivity to chemotherapy was observed to occur independently of the induction of autophagic processes [84].

#### 4.3.2. XIAP

XIAP can be indirectly suppressed by GAS5 and, in luminal breast cancer, is associated not only with apoptosis but also with autophagy [85].

#### 4.3.3. TRAIL

Though we already described how GAS5 expression can lead to TRAIL activation, TRAIL’s role in autophagy in breast cancer should be noted separately. The use of TRAIL-mediated apoptosis for the treatment of breast cancer is considered a promising method, although there are challenges to its use [86]. The chronic exposure of breast cancer cells to TRAIL induces increased autophagic activity, which protects breast cancer cells from TRAIL-driven apoptosis [87].

#### 4.3.4. PTEN

In breast cancer, the suppression of PTEN, the expression of which is enhanced by GAS5, attenuates not only apoptosis [67] but also autophagy [88].

#### 4.3.5. mTOR

mTOR is an important regulator of autophagy, including in breast cancer. The mTOR 1 complex inhibits the ULK1-Atg13-FIP200 complex, which is key for autophagosome formation [83]. The mechanisms of mutual suppression of GAS5 and mTOR expression are described above, although it should be noted that the suppressive effect of GAS5 on mTOR has, to date, been observed in several cancer types but not breast cancer.

### 4.4. GAS5 and Pathways in Breast Cancer

#### 4.4.1. PI3K/AKT/mTOR Pathway

The phosphoinositide 3-kinase (PI3K)/AKT/mTOR pathway is the most important oncogenic pathway altered in breast cancer. However, different components of this pathway are affected more often than others in different breast cancer subtypes. Breast cancer cells may or may not feature three important receptors: estrogen receptor (ER), progesterone receptor (PR), and human epidermal growth factor receptor 2 (HER2). PI3K/AKT is activated in cancer by several different mechanisms, including somatic activating mutations (e.g., PIK3CA and PIK3R1), the amplification of genes encoding key components (e.g., PIK3CA and AKT), and the amplification/overexpression of upstream receptor tyrosine kinase (e.g., insulin-like growth factor receptor 1, HER2) [89]. Typical mutational and epigenetic changes in individual genes in the PI3K/AKT/mTOR pathway are characteristic of specific breast cancer subtypes. Phosphatidylinositol-4,5-bisphosphate 3-kinase catalytic subunit alpha (PIK3CA) expression is very often increased in luminal A (ER+, progesterone receptor-positive or negative (PR±), HER2-) and HER2+ tumors [90]. In TNBC (ER-, PR-, HER2-), deletions occur frequently, leading to a loss of expression of PTEN and Inositol polyphosphate 4-phosphatase type II phosphatases. In these subtypes of breast cancer, the mechanism of activation of the PI3K/AKT/mTOR pathway is largely determined by genomic changes in these phosphatases [91,92]. In other subtypes of breast cancer, PTEN inactivation may be associated with promoter methylation, protein instability, or post-translational modification [93].

Agents targeting ER+ and HER2+ breast cancer are among the most successful therapies to date. However, initial or acquired resistance to these agents limits treatment options. Activation of the PI3K pathway in HER2+ breast cancer is responsible for resistance to anti-HER2 therapy. Activation of the PIK3CA mutation or a loss of PTEN was found to be sufficient to provide resistance to HER2-targeted therapy in preclinical models [94]. For TNBC, the absence of the amplification and/or overexpression of ER and HER2 significantly reduces the possibilities of therapy with TNBC due to a lack of targets. In these cases, the use of inhibitors (of various stages) of the PI3K/AKT pathway—particularly inhibitors of mTOR—is considered a promising approach, especially for the mesenchymal-like subset [89,95]. As already noted, the inhibition of mTOR also increases the expression of GAS5, which may be one of the additional mechanisms of action for GAS5. Intensive clinical studies on AKT inhibitors were performed in previous studies (e.g., [96,97]).

**Figure 3:**
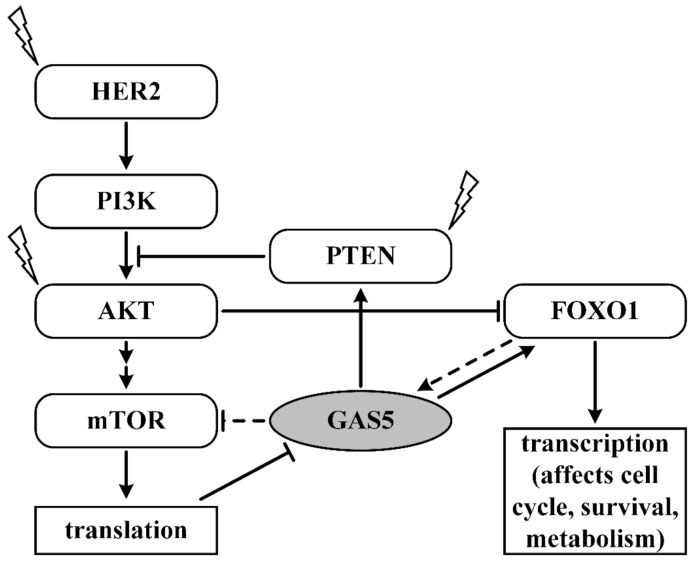
GAS5 and PI3K/AKT/mTOR pathway in breast cancer. Straight arrows: stimulation, blunt arrows: inhibitory effects, dotted line: estimated impact, lightning bolt: proteins often affected by mutations in breast cancer. Two consecutive arrows indicate other participants in the process, i.e., that the connection is not direct but occurs through intermediaries.

The action of GAS5 suppresses the effects of the PI3K/AKT pathway at several stages simultaneously (Figure 3). In particular cases, an increase in this pathway’s expression can affect breast cancer regardless of the mechanism of activation. GAS5 increases the expression of PTEN in several ways [67,68] and increases the expression of FOXO1 [54]. Moreover, in breast cancer, as in other types of cancer [27,29], the suppression of mTOR and GAS5 may be mutual. Current knowledge thus implicates GAS5 as a promising therapeutic target in breast cancer. Knowledge of its role is important to understand the mechanisms of breast cancer.

#### 4.4.2. Wnt Pathway

Dysregulation of canonical and non-canonical Wnt-signaling occurs in TNBC [98]. One of the mechanisms of this dysregulation may be a decrease in the expression of GAS5, which binds to miR-221-3p. MiR-221 inactivates DKK2, which inhibits the Wnt/β-catenin pathway in MCF-7 [53]. MiR-221/222 also targets other inhibitors of the Wnt/β-catenin pathway in TNBC [99].

#### 4.4.3. NF-κB Pathway

The activation of nuclear factor-kappa B (NF-κB), a pro-inflammatory transcription factor, is commonly seen in breast cancer. NF-κB activation promotes the development of a hormone-independent, invasive, high-grade tumor phenotype and is specifically associated with a particularly aggressive ER- and HER2+ breast cancer subtype known as inflammatory breast cancer. Another subtype of breast cancer with a high level of constitutively active NF-κB signaling is TNBC [100]. cIAP2 expression can be influenced by a riborepressor in GAS5 [36]. cIAP2 is involved in processes leading to the activation of NF-κB, and cIAP2 is also activated by NF-κB in breast cancer [58]. It was also reported that activation of the DR4 receptors by TRAIL can lead to activation of the NF-κB signaling pathway [101]. Thus, the influence of GAS5 on this pathway may be ambiguous.

#### 4.4.4. Signaling Pathways Regulating the Expression of GAS5

The expression of GAS5, similar to that of several other oncosuppressive lncRNAs, is inhibited by c-MYC, which is amplified in 15% of breast cancer cases [102]. Another negative regulator of GAS5 in breast cancer is Notch-1 [103].

## 5. Conclusions

LncRNA GAS5 is involved in the epigenetic regulation of genes, miRNAs, and proteins. GAS5 is downregulated in multiple cancers and acts as a tumor suppressor in breast cancer. In breast cancer, GAS5 participates in the activation of proteins that include PTEN, PDCD4, DKK2, FOXO1, and SUFU through a ceRNA mechanism mediated by various miRNAs, including miR-21, miR-222, miR-221-3p, miR-196a-5p, and miR-378a-5p. Thus, GAS5 contains multiple MREs for binding these oncogenic miRNAs in the upregulation of suppressor proteins in breast cancer. Some of these miRNAs, in turn, suppress the expression of GAS5, which generates positive feedback systems capable of “switching” the mode of cell functioning. The gene encoding suppressive lncRNA GAS5 harbors a promoter CpG island, and GAS5 expression level is also regulated epigenetically through the participation of promoter methylation in breast cancer. Importantly, through these interactions, lncRNA GAS5 may increase sensitivity to different drugs and enhance the chemotherapy treatment of breast cancer patients.

Many experimental and clinical studies have revealed the functions of GAS5 in the biological processes of breast cancer. The functions of GAS5 in breast cancer are well established in apoptosis (both spontaneous and induced by chemotherapy). In future studies, the role of GAS5 in other breast cancer processes should be studied in more detail.

GAS5 is a key player in the regulation of the PI3K/AKT/mTOR pathway. GAS5 influences various stages of this pathway and participates in the regulation of Wnt/β-catenin and NF-κB. Through epigenetic and other mechanisms, GAS5 can increase sensitivity to chemotherapy and improve prognosis. GAS5 is thus a promising target in the treatment of breast cancer patients. In particular, influencing the expression of GAS5 in vivo is a promising target for therapy. The expression of GAS5 is increased by mTOR inhibitors, which are now widely used in clinical practice and have been proposed to improve the responses to chemotherapy in breast cancer [104]. A meta-analysis of clinical trials in the treatment of solid tumors showed that the use of mTOR inhibitors can significantly increase progression-free survival [105]. Unfortunately, the clinical use of such inhibitors in breast cancer is hampered by significant toxicity [106]. Since GAS5 affects many processes in the body in addition to carcinogenesis (in particular, it increases insulin sensitivity), non-toxic and efficient cellular uptake molecules that can block GAS5’s UPF1-mediated degradation via the nonsense-mediated RNA decay pathway are being pursued [33].

## Data Availability

Not applicable.
